# Linguistic measures of chemical diversity and the “keywords” of molecular collections

**DOI:** 10.1038/s41598-018-25440-6

**Published:** 2018-05-15

**Authors:** Michał Woźniak, Agnieszka Wołos, Urszula Modrzyk, Rafał L. Górski, Jan Winkowski, Michał Bajczyk, Sara Szymkuć, Bartosz A. Grzybowski, Maciej Eder

**Affiliations:** 1 Institute of Polish Language, Polish Academy of Sciences, Cracow, Poland; 2 Institute of Organic Chemistry, Polish Academy of Sciences, Warsaw, Poland; 3 Center for Soft and Living Matter of Korea’s Institute for Basic Science (IBS), Ulsan, South Korea; 4 Department of Chemistry, Ulsan National Institute of Science and Technology, Ulsan, South Korea

## Abstract

Computerized linguistic analyses have proven of immense value in comparing and searching through large text collections (“corpora”), including those deposited on the Internet – indeed, it would nowadays be hard to imagine browsing the Web without, for instance, search algorithms extracting most appropriate keywords from documents. This paper describes how such corpus-linguistic concepts can be extended to chemistry based on characteristic “chemical words” that span more than traditional functional groups and, instead, look at common structural fragments molecules share. Using these words, it is possible to quantify the diversity of chemical collections/databases in new ways and to define molecular “keywords” by which such collections are best characterized and annotated.

## Introduction

**Figure 1:**
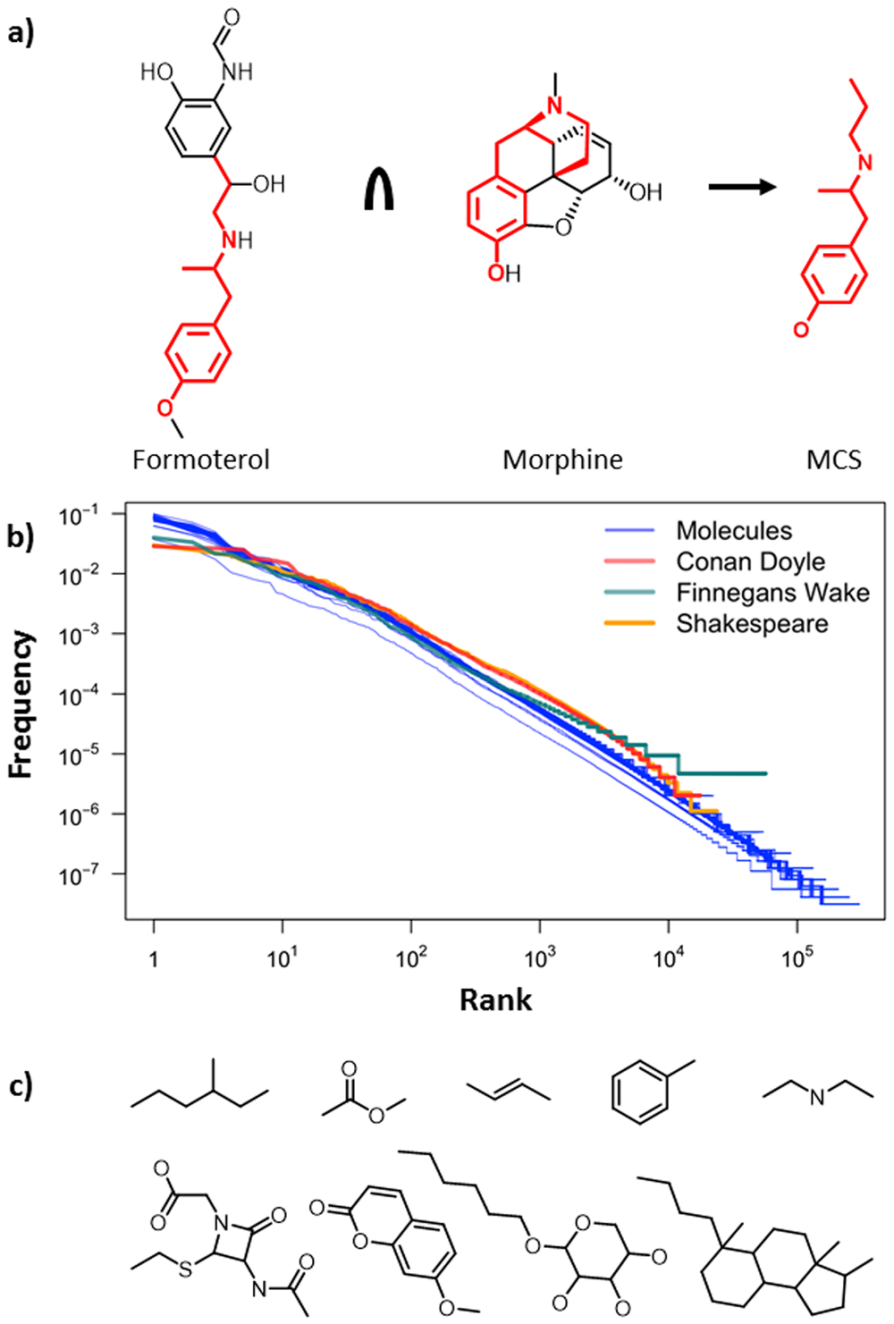
Chemical words and vocabularies. (**a**) Illustration of a common maximal substructure, MCS (colored red), between two molecules, formoterol (an anti-asthmatic/COPD drug) and morphine. (**b**) Blue lines are statistics of distinct MCS “words” for the entire 1.75-million-rich chemical vocabulary and over 100 randomly chosen subsets of Reaxys molecules (each subset with 500 to 9,000 molecules and 124,750–40,495,500 word tokens). The red, green, and orange lines are the distributions of words in, respectively, Conan Doyle’s collected works, Joyce’s “Finnegans Wake” novel, and Shakespeare’s works. All dependencies are rescaled by the number of words/molecules in a given set. As seen, the distributions for all sets are similar. (**c**) Examples of chemical words – those in the upper row are popular but not very specific fragments. Those in the lower row are less popular but immediately signal a specific group of chemicals (from left to right 1^st^ = penicillins and cephalosporins, 2^nd^ = coumarins, 3^rd^ = carbohydrates, 4^th^ = steroids). Note that the structures shown are molecular fragments not actual molecules with correct valences (e.g., if oxygen is monovalent, it can be attached to H, alkyl, aryl, etc.).

Searches for new drugs often begin with high-throughput screening of large molecular libraries^1–6^. In addition to meeting several criteria of drug-likedness^1,2,6^, it is desirable for such libraries to be structurally diverse – that is, to cover as much of the chemical space^7^ as possible and thus maximize the likelihood of finding a “hit” compound. Molecular diversity is typically quantified using various descriptors^6,8,9^ ranging from scalar parameters (molecular weight, solubility, numbers of specific types of atoms and/or bonds, measures of branching, etc.), to vectors accounting for the presence or absence of specific functional groups, to the so-called fingerprints describing molecular environments (subgraphs) of atoms within a molecule^10^. While the information about functional groups and atomic environments certainly reflects molecule’s chemical properties and connectivity, these measures are not necessarily the patterns by which organic chemists recognize and categorize specific molecules. For instance, we recognize progesterone and testosterone as belonging to the same class of steroids not by the presence and placement of individual OH or C=O groups or by considering the environments of every atom, but rather by the characteristic system of four fused rings common to both molecules. Accordingly, such common patterns – and in particular, maximum common substructures, MCS (Fig. 1a) – have long been considered useful in quantifying molecular similarity (or diversity)^11–14^ and are known to avoid many problems associated with measures based on Tanimoto-type coefficients (e.g., dependence on the fingerprint chosen, or molecule size^15,16^). Moreover, our own group has shown^17^ that the popularity-vs-rank distributions of MCS derived from mid-size sets of small molecules are power laws (a.k.a. Zipfian distributions) and similar to the corresponding distributions of words in English. This finding indicates that the MCS could be construed as counterparts of words in a natural language and that it should therefore be possible to apply to these chemical substructures the methods of computational linguistics^18,19^ which have proven so powerful in analyzing and interpreting large corpora of texts, and which have been of recent interest in the chemical sciences^20^. In the latter context, we previously used such methods to identify most information-rich bonds within molecules^17^ whereas, more recently, the team from IBM Zurich applied the concepts of chemical linguistics to the prediction of reaction outcomes^21^. Here, we build on the analogies between words in a natural language and the MCS “chemical words” (i) to formulate new, linguistic measures of chemical diversity over molecular libraries, (ii) to define a metric quantifying a library-to-library “distance”, and (iii) to use this metric to identify words that are most characteristic of a given library and can thus serve as its “keywords”. The usefulness of these chemical-linguistic measures is evidenced by the analyses in which sets of common chemicals, drugs, natural products, and commercial libraries of small molecules are compared and contrasted based on the “vocabularies” of MCS-words and are annotated in a chemically meaningful ways using MCS “keywords”.

## Methods

### “Chemical words”

Figure 1a illustrates the conceimpt of a “chemical word” based on the maximal common substructure, MCS, between a pair of molecules. When analyzing a collection of molecules, the MCS’s for all molecule pairs are calculated and their frequency of occurrence is plotted against the rank (i.e., the most popular MCS “word” has rank 1, the second most popular has rank 2, etc.). Importantly, in ref.^17^, we showed that when plotted on a doubly logarithmic scale, the dependencies of frequency vs. rank are linear and overlap with those characterizing English prose. In this initial work, we were able to analyze relatively small collections of molecules (<2,000) for which the numbers of pairwise comparisons was up to 2,000,000 and the number of distinct words was up to ca. 40,000. In Fig. 1b, we show that the linear log-log plots are also observed for much larger sets, up to 688,000,000 pairwise comparisons, and with the total number of distinct MCS “words” in our chemical “vocabulary” over 1,750,000. Figure 1c provides some examples of such chemical words. We note that these words span more than traditional functional groups and some comprise fragments indicative of a certain class of chemicals (e.g. in the lower row of Fig. 1c, penicillins and cephalosporins, steroids, carbohydrates, coumarins). Interestingly, when distributions of some other features of molecules are considered (e.g., frequencies of atoms, see Supplementary Fig. 1), they are generally not power laws, implying a special status of MCSs as the true “words” of chemistry.

### Molecular collections

With the chemical words defined as above, we performed linguistic analyses of various types of chemical collections: **(*1*)** a set of ca. 104,000 unique molecules chosen at random from the Reaxys repository (www.reaxys.com) for which we calculated (using RDkit, version 2015.09.2, http://www.rdkit.org/) 668,000,000 unique pairwise comparisons (chosen at random; 14% of the total possible five billion molecule-to-molecule comparisons); **(*2*)** multiple 1,000-molecular-long subsets of (1); **(*3*)** a set of 1,000 natural products chosen randomly from 1489 natural-product entries in the Zinc Database (http://zinc.docking.org/catalogs/specsnp); **(*4*)** 1,000 FDA-approved drugs chosen at random from 1,800 drugs deposited in https://www.drugbank.ca/; and **(*5*)** ten samples, 1,000 molecules each from the libraries sold by Mcule (www.mcule.com), a leading commercial provider of compound libraries. The “vocabularies” derived from these collections comprised 1.75 million unique MCS words for (1), and tens of thousands words for other, smaller collections.

## Results and Discussion

### Linguistic measures of chemical diversity

We first considered a diversity measure called a type-token ratio, TTR, which is used widely in corpus linguistics^22^ to quantify lexical morphological richness of a language^23^, improvement in writing skills^24^, or individual styles of authors^25^. In some languages, TTR does not depend on the text genre (e.g., in Czech^25^), in others the differences are pronounced, also between written and spoken language (e.g., in English^26^). TTR is simply the ratio of unique to the total number of words in a given text. For instance, the opening sentence of Arthur Conan Doyle’s “The Study in Scarlet” reads as follows: “*In the year 1878 I took my degree of Doctor of Medicine of the University of London, and proceeded to Netley to go through the course prescribed for surgeons in the army*.” This sentence comprises 32 words (“tokens”), of which 24 are unique “types” (since “the” and “of” repeat four times while “in” and “to” each occur twice), such that the TTR = 24/32 = 0.75.

**Figure 2:**
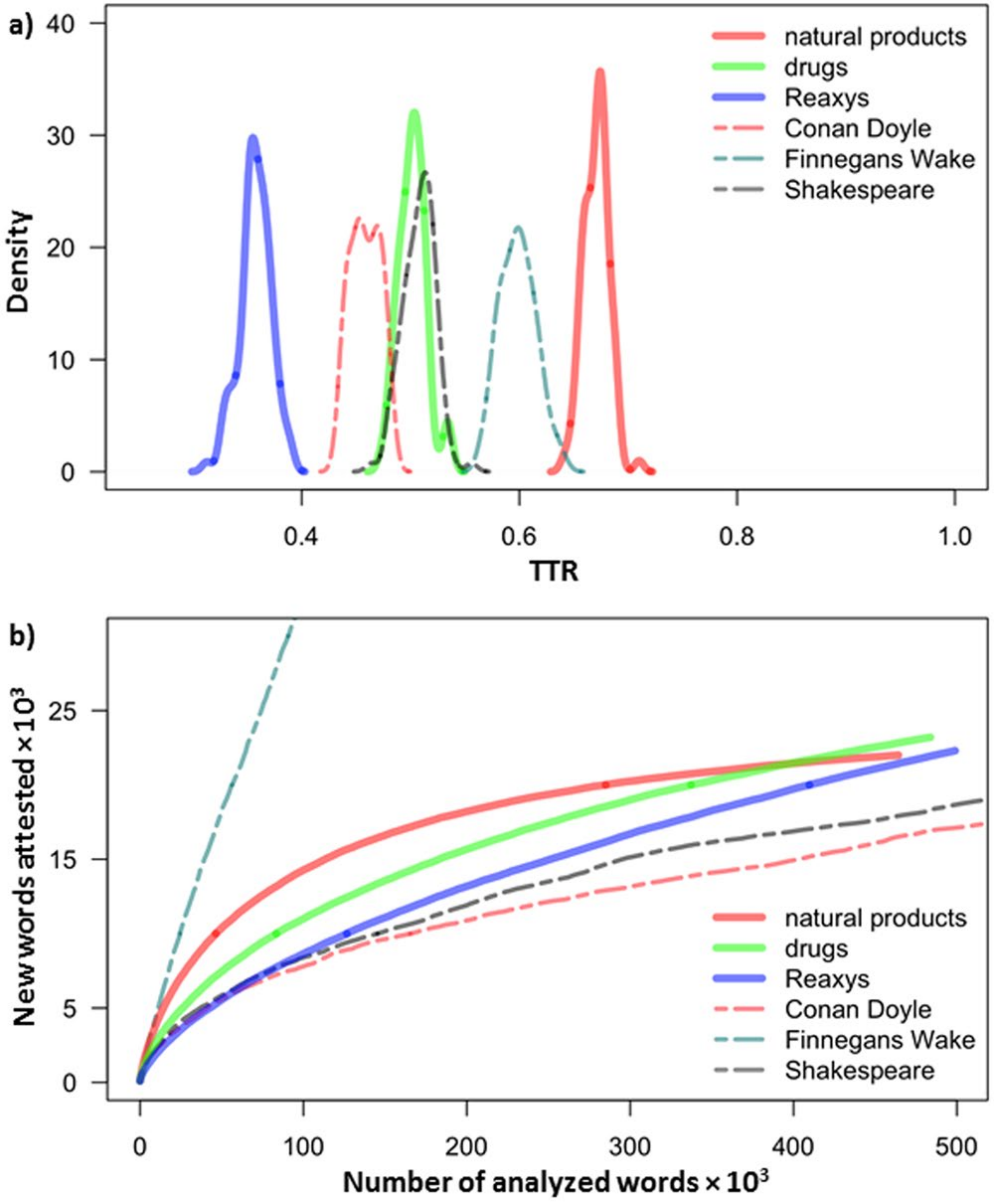
Measures of chemical diversity in chemistry and in literary prose. (**a**) Density (y-axis) of word windows with a given MWTTR-value (x-axis). MWTTR (Moving Window TTR) is computed by traversing the whole text with a window of a given length (here, 1,000 words). Whereas in literature the ordering of words matters, chemical collections do not have any meaningful ordering of molecules – here, the windows are sliding over molecules placed in random order (the results do not change upon reshuffling the molecules). Solid lines are for chemical collections whereas dashed lines are for literary works. (**b**) Heap’s law in language and in chemistry. The curves count the increase in the number of unique words (or MCS chemical words) encountered as one traverses a given collection. Solids lines are for sets of molecules, dashed lines are for literary works. Heap’s law for the chemicals does not depend on the choice or the order of the molecules in a collection (see Fig. S2 in the SI).

Our TTR analyses were performed using subsets of 50,000 words chosen randomly from larger vocabularies characterizing a given collection of molecules (for collection of size *n*, the vocabulary is comprised of *n*(*n* − 1)/2 MCSs derived from pairwise molecule-to-molecule comparisons). These analyses give TTR values of 0.1058 for randomly chosen molecules (collection **(*2*)**), with averaging done over 100 subsets), 0.2051 for the collection **(*3*)** of natural products, and 0.1469 for the collection **(*4*)** of drugs. Quite remarkably, this linguistic richness of chemical collections is commensurate with that of the works of Shakespeare (0.1296) and Conan Doyle (0.1228), but is lower than Joyce’s “Finnegans Wake” novel (0.3385), which is known to be a linguistic outlier with incredibly rich word inventory coming from numerous languages. Within chemistry, natural products are more diverse than drugs and both types of sets are more diverse than an equally-sized sample of molecules taken at random from Reaxys. In making such comparisons, however, it must be remembered that they remain strictly valid for the same lengths of the text samples. This is so because TTR is sensitive to and generally decreases with the length of the input text^18^ (as common words start repeating). One way around this problem is to divide the text into equal-size parts and then take an average over the TTRs of these parts. Another approach is to use moving averages, in which a “window” of a given length is moved over the text and the TTR scores are averaged over all window positions^27^. Kubát & Milička^25^ suggested that one can also calculate the distributions of the numbers of windows enclosing texts of a given TTR. We follow this Moving Window TTR, MWTTR, approach in Fig. 2a, which plots the distributions of TTR values within moving windows that are 1,000-words (for literary samples) or 1,000-chemical-words (for sets of molecules) long. As seen, the MWTTR measure preserves the ordering of simple TTR but (i) the differences between the samples are more spread-out and (ii) natural products are now more diverse than Joyce’s novel (see Supplementary Fig. 2 for further illustration of Finnegan’s linguistic uniqueness).

Based on the above considerations, we conclude that while various forms of TTR give qualitatively similar rankings of molecules’ diversity, certain differences between these measures exist – indeed, in linguistics, TTR alone is considered too simple a measure to provide reliable information about word distributions. Consequently, TTR is often supplemented by other metrics, especially those that plot the growth rate of unique vocabulary as a function of text length – that is, by curves plotting the number of new words (“word types”) one encounters while reading the text (comprised of “word tokens”). Such curves are described by the so-called Herdan’s law (also known as Heap’s law^28,29^), *V*_*R*_(*n*) = *Kn*^*β*^, where *V*_*R*_ is the number of distinct words in a text of size *n*, and *K* and *β* are free parameters that are determined empirically. Dashed lines in Fig. 2b trace vocabulary growth for the Joyce’s, Shakespeare’s, and Conan-Doyle’s works we considered before – as seen, for the latter two authors, the number of new words starts levelling off relatively early; in contrast, Joyce’s “Finnegans Wake” keeps surprising the reader with new vocabulary until the very end. Extending this representation to the words of chemistry (solid lines) and scanning through the vocabularies derived from our various chemical collections, we see that natural products and drugs show similar trends (though for the natural products, the rate of increase is initially steeper) whereas the vocabulary of common chemicals from Reaxys is more constrained. In other words, drugs and natural products are again more internally diverse than random chemicals. We emphasize that in all cases, the curves fit to the Heap’s law well, with the *R*^2^ values as high as 0.99 for linguistic corpora and 0.98 for chemical data.

These conclusions merit two additional comments. First, the curves for the chemical collections are insensitive to the order in which the molecules are “read” (Supplementary Fig. 3). Second, it should be remembered that when comparing collections of different numbers of molecules, the number of MCS words in their “vocabularies” will be different which, in turn, will affect the rate of increase. This is seen in Supplementary Fig. 4 where the more shallow, blue curve is for ~20,000 words derived from 1,000 molecules whereas the steeper, orange curve is for 1,750,000 words derived from collection **(*1*)** (based on 668,000,000 word-to-word comparisons). As in the case of TTR measures, it is therefore important to make comparisons between like-sized sets.

### Diversity within molecular libraries

**Figure 3:**
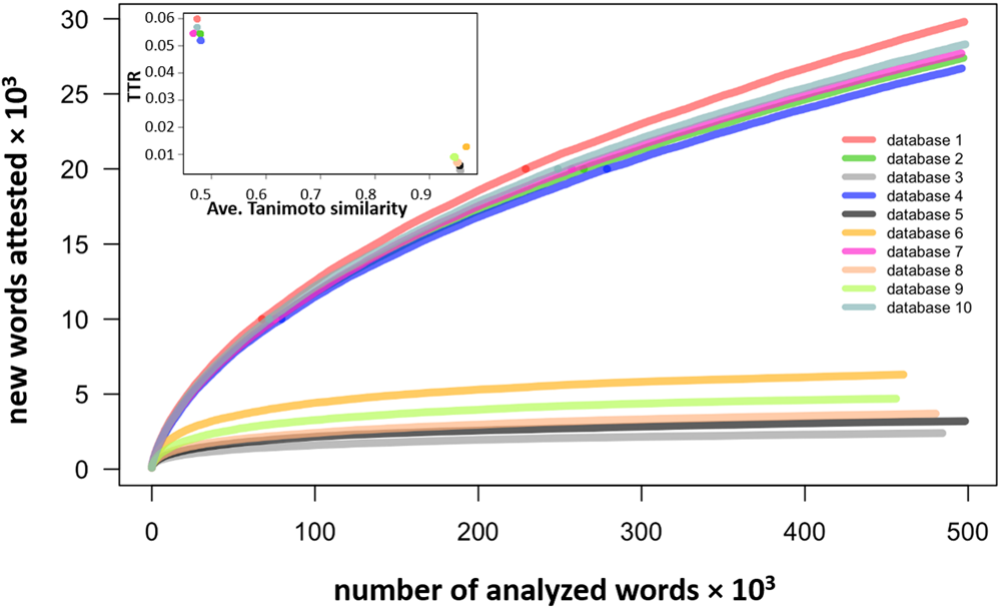
Linguistic measures of diversity in commercial chemical libraries. The main graph illustrates the Heaps’ law (i.e., the number of new MCS chemical words found when scanning through the “vocabulary” characterizing a particular molecule collection) for ten samples of commercial databases from the Mcule company. Each sample comprises 1,000 molecules and (1,000·1,000/2) − 1,000 = 499,000 pairwise comparisons between different molecules are made to derive its MCS vocabulary. Inset plots TTR values for each database against Mcule’s Tanimoto-based similarity measure.

Practically, the above measures can be used to estimate the diversity in chemical libraries and also visualize it in ways not available with traditional approaches based on Tanimoto coefficients (cf. a typical matrix of Tanimoto coefficients in Fig. S2 in ref.^17^). To illustrate this, we teamed up with the Mcule company (www.mcule.com) – a leading European provider of molecular libraries – who shared with us ten samples of their choosing, each 1,000 molecules, drawn from their commercial libraries of potential lead compounds. The exercise was structured as a blind test in the sense that we were initially not provided any information about the samples’ diversity. By plotting the rate of new chemical word increase (as in Fig. 2b), we readily established that the samples group into two families of similar diversities – five less diverse (set of five lower curves in Fig. 3a) and five significantly more diverse (upper five curves in Fig. 3a). We also characterized the samples by TTR values and found that for the first family of five samples, TTRs were between 0.024 and 0.069, whereas for the second family of five, between 0.150 and 0.164 (note: these values can be compared with the values of other collections discussed above since the vocabularies were similarly-sized). We then communicated these results to Mcule who, in turn, provided us with their own estimates of diversity. In the Mcule’s measure, popular in drug-discovery industry, each molecule was compared with other molecules in the set and assigned a maximum Tanimoto coefficient (e.g., a value of, say, 0.76 for a given molecule would mean that the closest analogue in a given set has a Tanimoto coefficient of 0.76 and all other molecules are less similar). When averaged over an entire collection, this measure decreases with increasing diversity. Figure 3b plots the values of our linguistic TTR metric against average Mcule values for each 1,000 molecule samples – as seen, the two measures correlate closely (R^2^ = 0.9545) though, again, only our approach allows for the visualization as in Fig. 3a.

**Figure 4:**
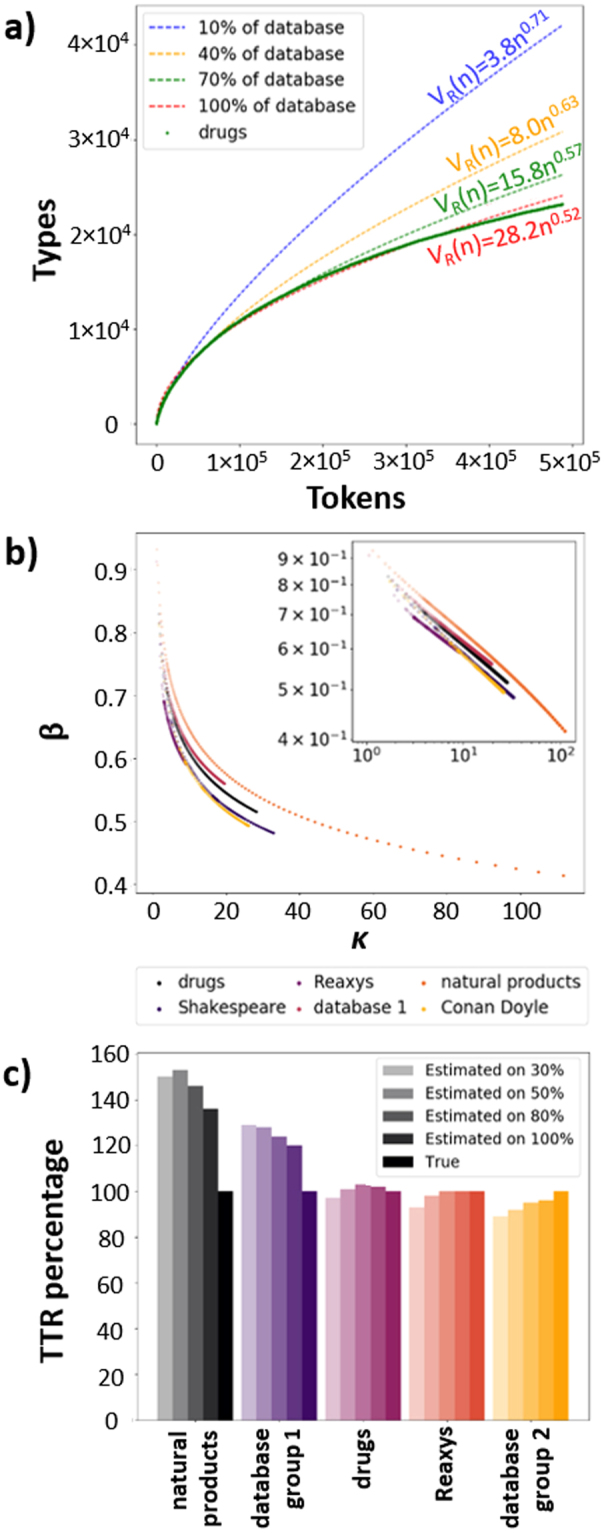
Estimation of linguistic richness bases on partial analyses of datasets and Heap’s law. (**a**) As progressively larger fractions of a particular collection/database (here, 1,000 drugs) are analyzed, the fits based on the Heap’s law, *V*_*R*_(*n*) = *Kn*^*β*^, converge to the type-token distribution characterizing the entire collection. (**b**) During such convergence, the exponents *β* decrease and prefactors *K* increase. The inset shows that this relationship is common to different molecular or literature collections – the straight lines on the doubly-logarithmic scale indicate a power law *β* ~ *K*^*−γ*^ (note: similar slopes correspond to similar values of *γ*). (**c**) Prediction of the type-token ratios, TTRs, based on the partial fits for different types of collections. The true value of the entire collection is taken as 100%. “Database group 1” and “database group 2” are the two families of Mcule databases from Fig. 3. The largest discrepancy between fits and real diversity is observed for natural products whose linguistic peculiarity is also manifest in our other analyses (cf. Figure 2b where the natural-products curve intersects dependencies for drugs and Reaxys molecules). For other collections, estimating 30–50% of the content already gives decent estimates of their actual diversity.

Another potential advantage over Tanimoto-based averages is that we can estimate the diversity of a molecular collection based on the analysis of only its subset. Recall that the curves such as those in Figs 2b and 3a fit well to the Heap’s law, *V*_*R*_(*n*) = *Kn*^*β*^. Such functional dependencies are also observed when analyzing portions (say, 30% or 70% of all molecules) within a collection. With the increasing size of this subset, the fits converge to the distribution characterizing the entire library (Fig. 4a). Importantly, we have verified that this convergence is similar for different datasets we studied – in particular, as the size of the subset under study increases, the best fits are such that prefactor *K* and exponent *β* are related by a power law *β* ~ *K*^*−γ*^ (Fig. 4b). Knowing this universal behavior, we can then extrapolate relatively well the diversity of the entire collection by analyzing only its subset (Fig. 4c) – in other words, we can significantly reduce the numbers of molecule-to-molecule comparisons (e.g., by a factor of 4 if 50% of the collection is taken) yet still obtain decent estimates of diversity. We note that this type of extrapolation cannot be used for Tanimoto-based methods for which no universal scaling with dataset size is neither known nor should generally be expected.

### Distributions of chemical words

**Figure 5:**
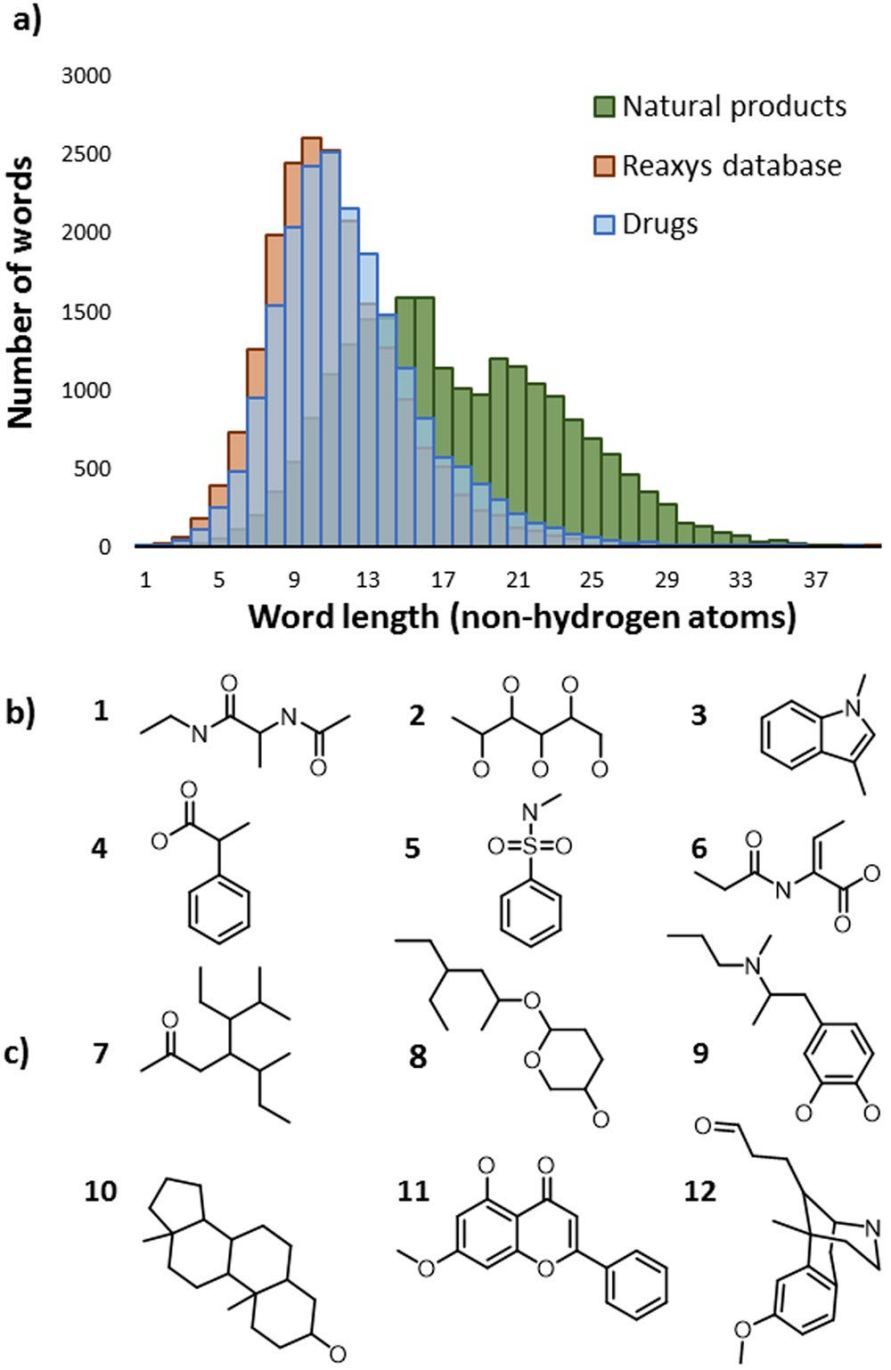
Distributions of MCS chemical word lengths and examples of such words. (**a**) Distribution of word lengths, measured by the number of non-hydrogen atoms, for molecules randomly chosen from Reaxys (orange), for drugs (blue), and for natural products (green). (**b**) Examples of words from the distribution of (**b**) drugs and (**c**) natural products. As in Fig. 1, the structures shown are molecular fragments not actual molecules with correct valences (e.g., if oxygen is monovalent, it can be attached to H, alkyl, aryl, etc.).

A potentially quite informative feature of chemical “vocabularies” is the nature of words they contain. For example, although drugs and natural products exhibit similar richness of vocabulary, one might suspect that the “words” in the two sets are of different lengths. Figure 5a plots the distribution of “lengths” (measured as the number of constituent non-hydrogen atoms) of unique MCS words derived from the randomly-chosen molecules in set **(*1*)**, drugs in set **(*3*)** and natural products in set **(*4*)**. As seen, the words in random molecules and in drugs are relatively short (maximum of the distribution at around 10 atoms) and distributed similarly. Examples of drug-based words in Fig. 5b correspond to structural fragments found in polypeptide antibiotics (e.g., bacitracin, dactinomycin; fragment 1 in the figure), glycoside containing drugs (e.g., amikacin antibiotic, topiramate anticonvulsant, 2), indole alkaloids (e.g., vincanire, ondansetron; 3), nonsteroidal anti-inflammatory drugs (e.g., ibuprofen, ketoprofen; 4), medications for erectile dysfunction or sulphonamide antibiotics (e.g., sildenafil, sulfacetamide; 5), and cephalosporin antibiotics (e.g., cefadroxil, cefazolin; 6). In contrast, an analogous distribution for natural products is much broader and features the main peak centered around 15–16 atoms and a “satellite” peak centered around 20 atoms. As illustrated in Fig. 5c, longer words occurring in natural products usually can be easily recognized as characteristic scaffolds of certain classes of compounds (e.g., steroids, 10, flavonoids, 11, or opioids, 12). Shorter words are generally less informative (e.g., structure 7) but in some cases can be recognized as substructures of classes such as sugars, 8, or catecholamines, 9.

### Characteristic chemical “keywords”

**Figure 6:**
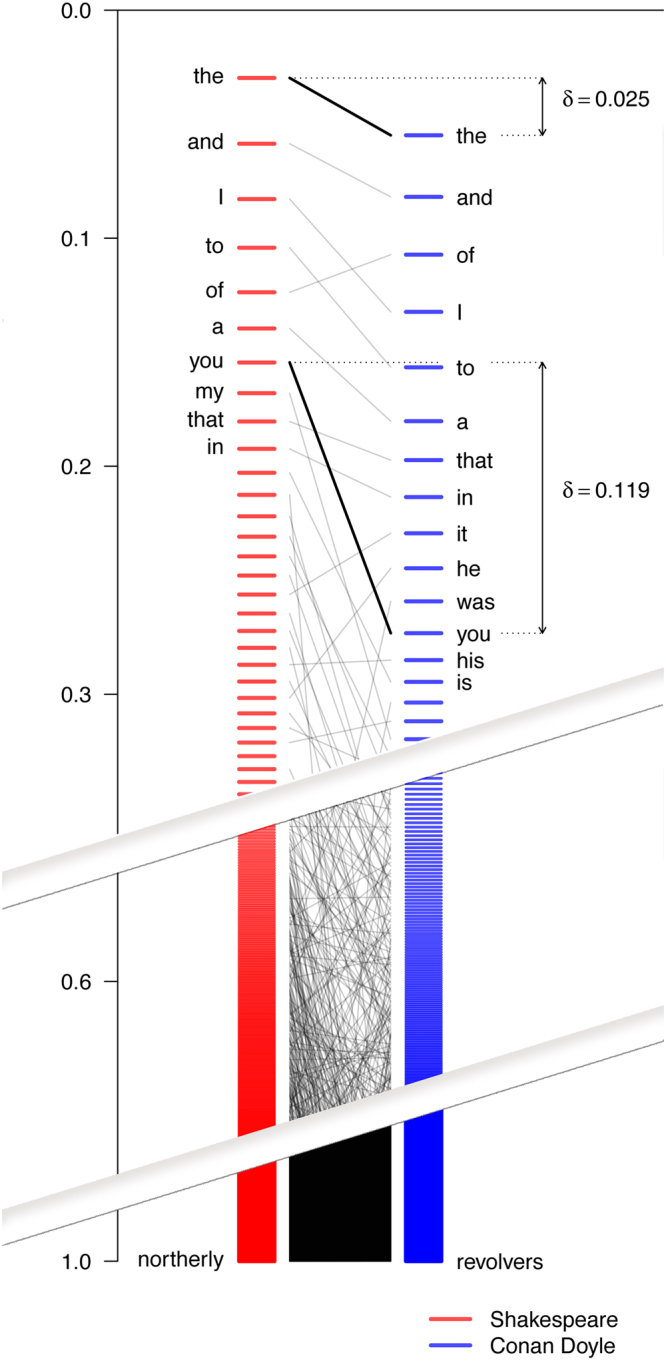
Frequency ranking and distances between words in literary prose. Differences in “positions” – defined as a cumulative sum of their relative frequencies – between words derived from the works of Shakespeare and Conan Doyle. The most popular words are at the top of the list and near 0.0 values. The position of the first word is equal to its relative frequency. Delta values measure the differences in words’ positions in the two lists.

Examples of chemical words in a given set of molecules prompt our final question – namely, how to determine quantitatively those words that are most characteristic of a given collection of molecules and could thus serve as its “keywords”. To do so, it is necessary to first develop a metric that measures a “distance” between molecules or sets of molecules. In linguistics, the efforts to compare two texts/corpora^30^, date back to 1950s but the existing measures are too simplistic or altogether not suitable for meaningful comparisons of chemical data. For instance, when a popular measure proposed by Kilggariff^31^ (Chi-by-degrees-of-freedom) is applied to our molecule collections, the distances from drugs, natural products, or other like-sized subsets of randomly-chosen Reaxys molecules to the larger Reaxys collection **(*1*)** are all similar (respectively, 1143, 1036, 1077–1087). Accordingly, we considered several other metrics ultimately focusing on the one based on the frequency-corrected positions of “chemical words” in ranked lists (i.e., sorted from the most to the least popular words). Specifically, consider a set of *N* “chemical words” ranked according to their frequency of occurrence in a given corpus/collection. Denoting the rank of word *x* as *r*(*x*) and its frequency as *f*(*x*), we can define the normalized position, *P*, of this word by summing up the frequencies of this and all words with lower ranks (i.e., more popular than *x*) as ${P}_{r(x)}=\frac{1}{N}\sum _{i=1}^{r(x)}f(i)$. Then, the distance between the same word in two sets of molecules, say A and B, can be defined as ${\delta }_{x,AB}=|{P}_{r(x),A}-{P}_{r(x),B}|$ (see also Fig. 6). Similarly, the distance between the entire two sets can be defined as an average of the word-to-word distances ${\delta }_{AB}=\frac{1}{{N}_{A}}\sum _{j=1}^{{N}_{A}}{\delta }_{x,AB}(j)$. We note that if a word is present only in one list (say, A) and absent in the other (B), we assign the maximal distance possible, $1-{P}_{r(x),A}$, as if the missing word were added at the very end of list B. We also observe that an important and appealing feature of this distance metric based on ranked MCS word lists is that it does not depend on the ordering of the molecules in either of the libraries being compared (which would be the case if pairwise comparisons were made between molecules in different libraries).

**Figure 7:**
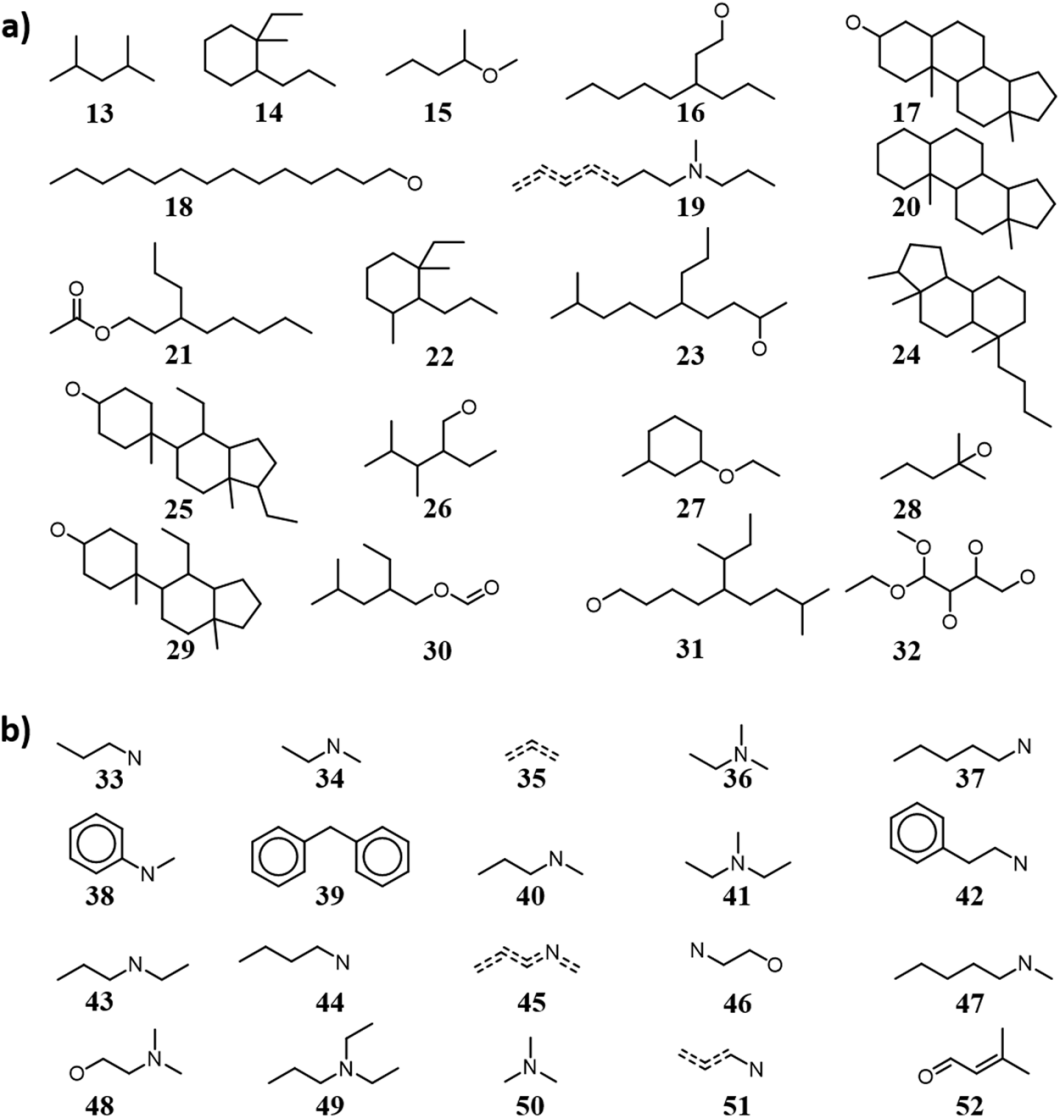
Top-20 MCS chemical “keywords” characteristic of (**a**) natural products and (**b**) drugs.

In implementing these ideas, we take the set *R* of molecules chosen randomly from Reaxys (collection **(*1*)**) as a chemical “universe” and a reference, and calculate the distances of molecules in other collections to this reference – for instance, the natural products are more distant (${\delta }_{AR}\,=\,$0.073) from this reference than drugs $({\delta }_{AR}\,=\,0.034)$. Importantly, with this metric, we can also identify the “keywords” in our collections of interest as the words having the largest distance to the reference collection – that is, those that are most distinct from the “random” molecules in Reaxys. For example, Fig. 7a shows 20 most characteristic “chemical keywords” of natural products. Keywords 17, 20, 24, 25, and 29 are immediately recognized as steroid scaffolds, 32 as part of carbohydrates, 19 of opioids, and 30, though less obvious perhaps, is a popular motif present in spirolactosteroids, Taxol, or vincristine. Keywords 13, 14, and 22 are indicative of fused aliphatic ring systems of steroids (13, 14, 22) and are also found in labdane diterpenes (13, 14, 22), tetracyline antibiotics (13) and secosteroids (13). Keywords containing branched and unbranched alkyl chains connected with oxygen by a single bond (15, 16, 18, 21, 23, 26, 27, 28, 31) might not seem as distinctive but they are, in fact, substructures of many classes of natural products including steroids (16, 18, 21, 23, 26, 28, 31), tetracycline antibiotics (16, 28, 31), anthracyclines (15, 27, 28), macrocyclic antibiotics (15, 18, 28), opioids (15, 16, 23, 27), fatty acids (18), flavonoid glycosides (15), catechins (15), or sesquiterpene lactones (21). Similarly, Fig. 7b gives top-20 drug-specific keywords which are, generally, simple substructures present in various classes of drugs. For instance, diphenylmethane motif, 39, is found in benzodiazepines, tamoxifen, ketoprofen or methadone whereas motifs 42, 46, and 48 are present in neurotransmitters (adrenalin, noradrenalin, 46, or dopamine, 42), β-lactam antibiotics (46, 48), tetracycline antibiotics (46, 48) or amino acids (46), and in multiple phenethylamines (amfepramone, salbutamol, 42). The α,β-unsaturated carbonyl 52 is characteristic of two classes of steroids (progestogens and corticosteroids) and cephalosporins. Tertiary amine motifs 36, 41, 49, 50 are present in the first and second-generation antipsychotic drugs (droperidol, aripiprazole, haloperidol), anticancer therapeutics directed against EGFR (gefitinib, brigatinib) and antimalarial medications (quinine, chloroquine, halofantrine). Secondary amine motifs 34, 40, 43, 47 are present in SSRI drugs (paroxetine, fluoxetine) and β adrenoreceptor antagonists (isoprenaline, fenoterol). Primary amine motifs 33, 37, 44 are found in amino acids (lysine, leucine) and anti-malarial drugs (tafenoqine, primaquine) whereas aniline motifs 38 and 51 are parts of benzodiazepines (diazepam, alprazolam) and tricyclic antidepressants (desipramine, lofepramine). The N-heterocycle motif 45 is present in quinoline alkaloids like camptothecin, proton-pump inhibitors (lasonoprazole or pantoprazole), and nucleoside reverse transcriptase inhibitors (abacavir or entecavir) used in anti-HIV/AIDS treatments.

## Conclusions

In summary, we have extended the concepts of linguistic similarity to collections of molecules. The measures we propose provide alternative and complimentary means of assessing chemical diversity and also visualizing it (cf. Fig. 3) in ways that are not possible with traditional Tanimoto-based approaches. This being said, we see the main value of our chemical-linguistic approach in annotating chemical collections with characteristic “keywords” by which such collections can be then searched/navigated, akin to searches of web documents/texts. In the business of small molecule libraries, chemical keywords could be used to discern sets of molecules most resembling specific classes of drugs. If additional, higher-order linguistic considerations – e.g., collocations of words “travelling together” or “avoiding each other” during chemical reactions – were taken into account, they could provide more information not only about characteristic structural features but also characteristic reactivity patterns^32,33^. In all such analyses, the “rate-determining” step is the extraction of vocabularies characterizing a given collection of molecules (entailing large numbers of molecule-to-molecule comparisons; e.g., 2.5 billion for a typical^34^ molecular library of ~100,000 compounds). Such calculations, however, can be accelerated by extrapolations based on the Heap’s law and are performed only once for a given set, and with modern computing resources can be completed within hours to days – all subsequent “keyword” comparisons/searches can then follow on much shorter time-scales.

### Data availability

Data, including vocabularies of MCS words, and computer codes that support the findings of this study are available from the corresponding author upon request.

## Electronic supplementary material

Supplementary Info
