# Supplementary material for: Linguistic measures of chemical diversity and the “keywords” of molecular collections
Source: Sci Rep. 2018 May 15;8:7598. doi: 10.1038/s41598-018-25440-6 (PMC5953938; doi:10.1038/s41598-018-25440-6)
Supplement: Supplementary file 1 — Supplementary Info [file 41598_2018_25440_MOESM1_ESM.pdf]

Supplementary Information for Manuscript titled *"Linguistic measures of chemical diversity and the "keywords" of molecular collections"* by Michał Woźniak, Agnieszka Wołos, Urszula Modrzyk, Rafał L. Górski, Jan Winkowski, Michał Bajczyk, Sara Szymkuć, Bartosz A. Grzybowski*, Maciej Eder*

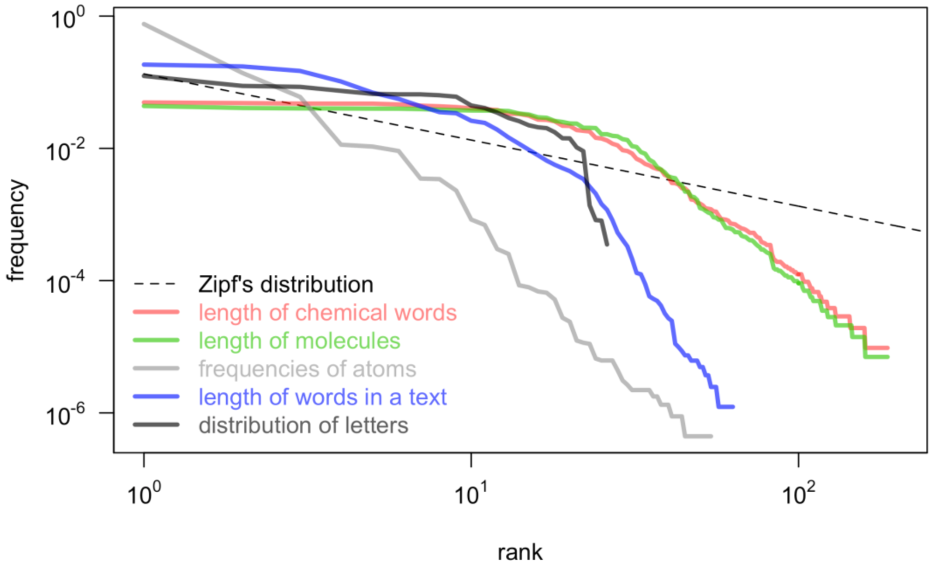

Supplementary Figure 1. Examples of non-Zipfian distributions. Neither the length of chemical MCS words, nor the lengths of molecules (both measured in characters in SMILES notation), nor the frequencies of atoms in molecules follow a power law. Similarly, in literary prose, the distributions of word lengths or of alphabet letters (tested here on Shakespeare corpus) are also not Zipfian.

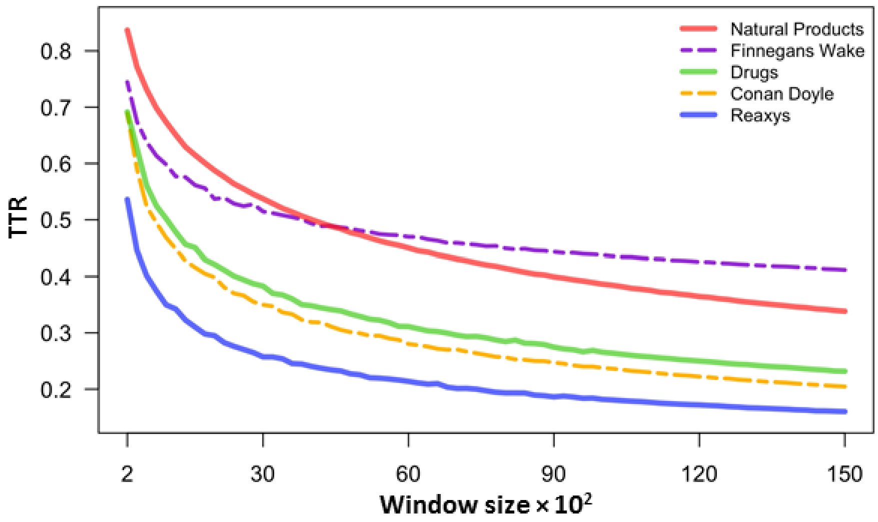

Supplementary Figure 2. Differences in the TTR diversity values (see main text) plotted as a function of the size of "windows" sliding along the text or collection of MCS chemical words. In general, the larger the window, the smaller the TTR diversity. For most texts/collections their relative diversity does not change – that is, the curves do not cross. One notable exception is "Finnegans Wake" which is slightly less diverse than natural products for small widows but remains diverse when window size increases (i.e., its TTR decreases only slowly). As a consequence, the curve for "Finnegans Wake" crosses that of natural products. This is yet another manifestation of Joyce's novel having extremely rich lexical diversity, even at larger lengths of fragments being probed.

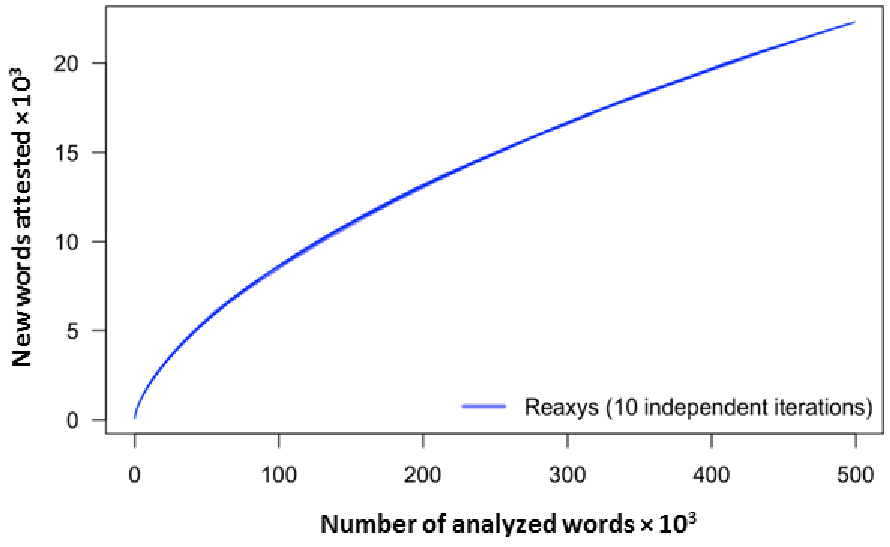

Supplementary Figure 3. Heap's law for the chemicals does not depend on the choice or the order of the molecules in a collection. Shown here are nine curves corresponding to nine random samples, each 1000 chemical words, taken at random from Reaxys database. All curves are virtually identical.

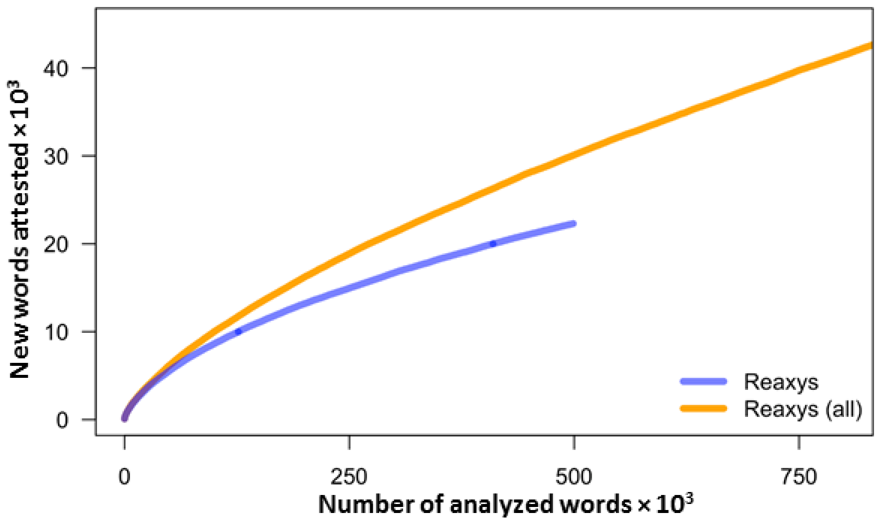

Supplementary Figure 4. Heaps' law for Reaxys subset from collection (2) and for Reaxys collection (1) (marked "all"). Blue curve shows the increase of word types with respect to word tokens in the subset of Reaxys database (499,500 chemical words out of 1,000 molecules). Orange

curve plots the same quantity but for 668,000,000 chemical words derived from 104,000 molecules.
